# Primary Diffuse Leptomeningeal Melanomatosis in a Child with Extracranial Metastasis: Case Report

**DOI:** 10.3390/curroncol31010041

**Published:** 2024-01-20

**Authors:** Shubin W. Shahab, Prabhumallikarjun Patil, Jason R. Fangusaro, Brooke Patteson, Adam Goldman-Yassen, Bree R. Eaton, William Boydston, Matthew Schniederjan, Dolly Aguilera

**Affiliations:** 1 Aflac Cancer and Blood Disorders Center, Atlanta, GA 30342, USA; jfangus@emory.edu (J.R.F.); brooke.patteson@choa.org (B.P.); dolly.aguilera@choa.org (D.A.); 2 Children’s Healthcare of Atlanta, Atlanta, GA 30322, USA; pspatil@emory.edu (P.P.); aegold5@emory.edu (A.G.-Y.); brupper@emory.edu (B.R.E.); william.boydston@choa.org (W.B.); matthew.schniederjan@choa.org (M.S.); 3 Department of Pediatrics, Emory University School of Medicine, Atlanta, GA 30322, USA; 4 Winship Cancer Institute, Atlanta, GA 30322, USA; 5 Department of Radiology, Emory University School of Medicine, Atlanta, GA 30322, USA; 6 Department of Radiation Oncology, Emory University School of Medicine, Atlanta, GA 30322, USA; 7 Department of Neurosurgery, Emory University School of Medicine, Atlanta, GA 30322, USA; 8 Department of Pathology, Emory University School of Medicine, Atlanta, GA 30322, USA

**Keywords:** primary CNS melanoma, meningeal melanomatosis, diffuse leptomeningeal melanoma, immunotherapy, MEK inhibitor

## Abstract

Primary meningeal melanomatosis is an extremely rare tumor with very few documented responses to treatment. A 3-year-old male with a complex past medical history, including prematurity and shunted hydrocephalus, was diagnosed with primary meningeal melanomatosis with peritoneal implants. Molecular testing revealed an NRAS Q61R mutation. The patient received proton craniospinal radiation followed by immunotherapy with nivolumab (1 mg/kg) and ipilimumab (3 mg/kg) IV every 3 weeks and, upon progression, he was switched to a higher dose of nivolumab (3 mg/kg IV every 2 weeks) and binimetinib (24 mg/m^2^/dose, twice a day). The patient had significant improvement of CNS disease with radiation therapy and initial immunotherapy but progression of extracranial metastatic peritoneal and abdominal disease. Radiation was not administered to the whole abdomen. After two cycles of nivolumab and treatment with the MEK inhibitor binimetinib, he had radiographic and clinical improvement in abdominal metastasis and ascitis. He ultimately died from RSV infection, Klebsiella sepsis, and subdural hemorrhage without evidence of tumor progression. This is the first report of a child with primary meningeal melanomatosis with extracranial metastatic disease with response to a combination of radiation, immunotherapy and MEK inhibitor therapy.

## 1. Introduction

The melanocytic tumors in the central nervous system (CNS) have been subdivided into four entities in the 2021 World Health Organization (WHO) classification of CNS tumors [1]. The most aggressive forms are meningeal melanoma and meningeal melanomatosis, its diffuse form. Meningeal melanoma is estimated to be extremely rare, with 0.005 cases per 100,000 [2] or about 1% of all melanoma cases [3]. Prognosis is generally poor, with an average life expectancy of approximately 22 months [4]. The outcome is worse for patients with diffuse leptomeningeal involvement, with life expectancy of approximately 4 months [5]. Only a handful of cases have been reported in children to date (see [6] and Table 1). We present a case of a young child with a complex past medical history diagnosed with diffuse leptomeningeal primary CNS melanomatosis with metastasis to the abdomen via ventriculoperitoneal (VP) shunt and subsequent response to therapy.

## 2. Case Presentation

**Figure 1:**
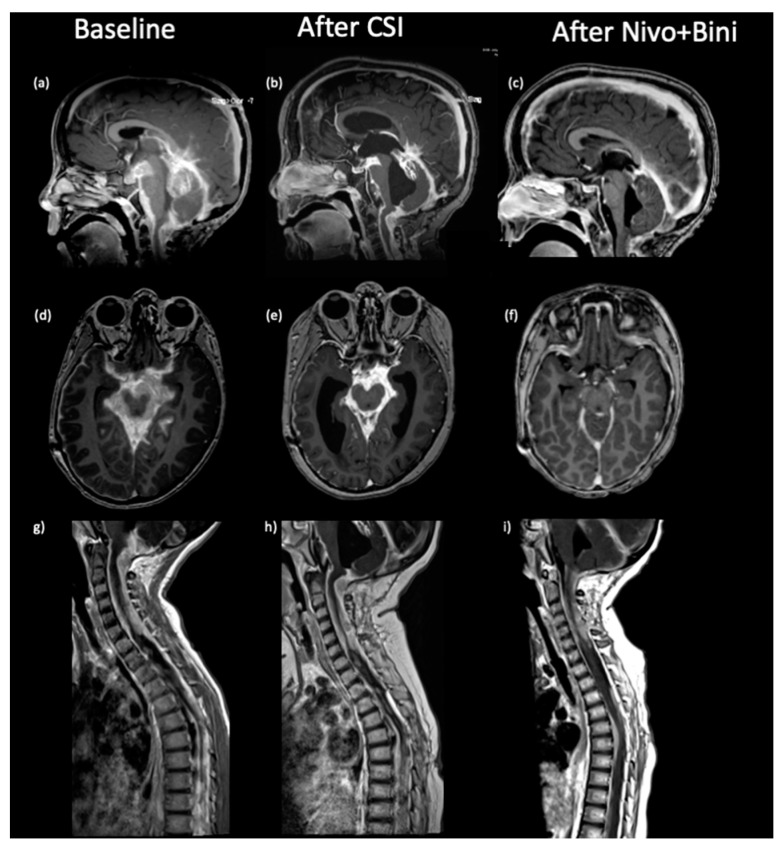
Sagittal (**a**–**c**) and axial (**d**–**f**) contrast-enhanced T1-weighted images of the brain and upper spine (**g**–**i**). At baseline, the brain demonstrated extensive enhancing leptomeningeal tumor with associated mass effect (**a**,**d**), as well as diffuse spinal leptomeningeal tumor, compressing the cord (**g**). Follow-up scans demonstrate decreased leptomeningeal enhancement in the basal cisterns (**b**,**c**,**e**,**f**) with relatively stable disease in the spine (**h**,**i**).

The subject is a three-year-old male who presented with a 1-week history of nausea, vomiting, and ataxia. He had a complex past medical history including the following: born prematurely at 25 weeks of gestation, autism spectrum disorder, sleep apnea, Chiari I malformation, and cystic fibrosis transmembrane conductance regulator-related metabolic syndrome. As an infant, he also required ventriculoperitoneal (VP) shunt placement for post-hemorrhagic hydrocephalus, Chiari decompression, and syringo-pleural shunt secondary to progressive cervicothoracic syringomyelia. At the initial presentation, he had headaches, ataxia, and vomiting. Non-contrast head-computed tomography (CT) showed no evidence of VP shunt malfunction or mass, and he was discharged home. However, he returned the next day with worsening ataxia, and contrast brain magnetic resonance imaging (MRI) revealed a dominant mass measuring 29 × 23.9 mm near the left foramen of Luschka as well as diffuse leptomeningeal enhancement throughout the brain and spine. (Figure 1a,d,g). Of note, a non-contrast MRI obtained 4 months prior to presentation did not show any evidence of mass lesion. He underwent a cervical laminectomy with biopsy of the subarachnoid tissue. 

**Figure 2:**
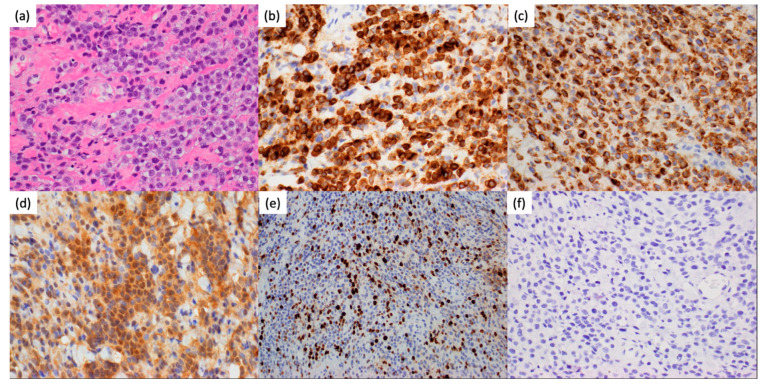
Histopathologic examination of the lesion revealed multiple pink-tan to white-tan soft tissue with sections demonstrating a collagenous background containing round epithelioid cells with moderate amounts of cytoplasm and round regular nuclei containing pale vesicular chromatin and prominent eosinophilic nucleoli as shown in (**a**) H&E stain. Corresponding (**b**) MelanA, (**c**) HMB-45, (**d**) S100, (**e**) Ki67, and (**f**) PD-L1 are shown. All images are taken at 400×, except for Ki67 imaged at 200×.

**Figure 3:**
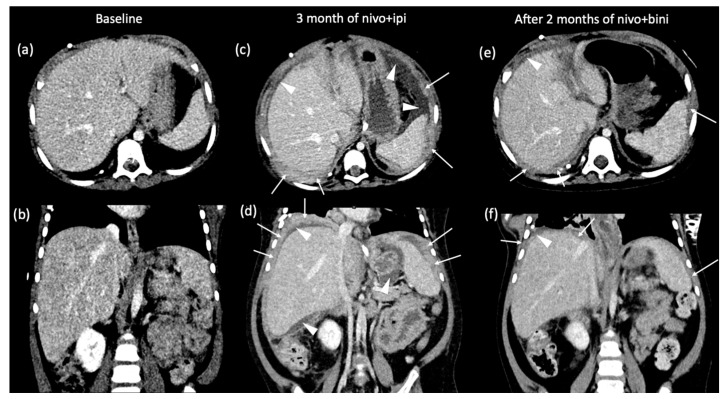
Axial (**a**) and coronal (**b**) contrast-enhanced CT of the abdomen at baseline are normal except for hepatomegaly. Axial (**c**) and coronal (**d**) contrast-enhanced CT of the abdomen after 3 cycles of nivolumab/ipilimumab demonstrating enhancing soft tissue along the peritoneum (arrows), compatible with peritoneal carcinomatosis, as well as moderate amount of ascites (arrowheads). Axial (**e**) and coronal (**f**) contrast-enhanced CT of the abdomen demonstrate significant improvement in ascites (arrowheads) and carcinomatosis (arrows) after 2 cycles of nivolumab/binimetinib.

The histopathologic exam of the biopsy specimen demonstrated sections with numerous apoptotic nuclei, mitotic figures with tumor cells positive for Melan-A, HMB-45 and S100 (Figure 2a–d), and negative for BRAF VE1. Ki-67 was elevated around 10–15% (Figure 2e). A PD-L1 stain was negative (Figure 2f). Molecular profiling of the tumor revealed an NRAS Q61R mutation with no other alterations, including no BRAF V600E mutation. The final diagnosis was meningeal melanomatosis. Cerebrospinal fluid (CSF) cytology from VP shunt was also positive for malignant cells. There was no evidence of skin or retinal lesions, suggesting a diagnosis of primary meningeal amelanotic melanomatosis. An abdominal CT demonstrated only hepatomegaly; positron emission tomography (PET)/CT showed implants on the bowel and peritoneal wall, concerning for abdominal dissemination through the VP shunt (Figure 3). The patient was initially treated with proton craniospinal irradiation. He received a total of 30 gray (Gy) relative biological effectiveness (RBE) in 10 fractions to the craniospinal axis. Sites of bulky nodular disease in the posterior fossa, cervical and upper thoracic (through T3) spine received an additional boost of 6 Gy (RBE) over two fractions, bringing the cumulative dose to 36 Gy (RBE).

Disease evaluation at the end of radiation demonstrated improvement of the disease burden (Figure 1b,e,h) in the brain and spine; however, CT scan demonstrated progression of intra-abdominal implants (Figure 3b). Systemic treatment was initiated with nivolumab (1 mg/kg IV every 3 weeks) and ipilimumab (3 mg/kg IV every 3 weeks).

After four cycles, the patient was noted to have continued increase in abdominal girth, worsening of ascites, as well as pleural effusion attributable to underlying metastatic lesions. Due to respiratory distress, a peritoneal and a right pleural drain were placed. Follow-up MRI of his brain and spine at this time revealed continuous improvement in CNS disease, but a CT abdomen and PET demonstrated significant worsening of abdominal disease (Figure 3). Pathologic exam of the pleural and peritoneal fluid confirmed the presence of malignant cells. Radiation to abdomen was deferred as the toxicity outweighed the benefits. Treatment was switched to the mitogen-activated protein kinase-kinase (MEK) inhibitor binimetinib (24 mg/m^2^/dose twice daily) with continuation of maintenance nivolumab at a higher dose and shorter interval (3 mg/kg IV every 2 weeks). Subsequent MRI after two cycles of this regimen demonstrated continued improvement of the CNS disease (Figure 1c,f,i), and abdominal CT demonstrated decreased peritoneal implants and improvement of ascites (Figure 3 and Supplementary Table S1). He continued his maintenance therapy given the response. Unfortunately, 7 months from initial diagnosis, the patient died from a combination of respiratory syncytial virus (RSV) infection, Klebsiella sepsis, and subdural hemorrhage.

## 3. Discussion

Primary CNS melanomatosis is an extremely rare entity. In many cases, there are delays in the diagnosis given that imaging findings are not characteristic. The differential diagnoses include neoplastic meningitis, CNS tuberculosis, and lymphoma, among others [16]. In the pediatric population, we only found 10 cases of pediatric primary CNS melanomatosis/melanocytosis, although we are unable to discern between the two, as most cases do not have pathologic details. Only one of these cases was amelanotic [10] (see Table 1). Median age was 11 years (range 2–17); six were male and four were females. None of the patients were long-term survivors, and most patients died within a year of diagnosis (range 2–18 months). Treatment varied widely, likely due to the rarity of these cases and lack of consensus on the ideal treatment regimen. Radiation was used in four patients and chemotherapy in six patients. Two patients were treated with immunotherapy (one with pegylated interferon and another with an MEK inhibitor followed by ipilimumab and nivolumab). For extracranial cases, amelanotic melanoma typically has a worse prognosis than pigmented melanoma [17], possibly related to delayed diagnosis and degree of cellular differentiation; however, the numbers are too small in CNS cases to predict whether amelanotic leptomeningeal melanomatosis has a worse outcome.

In a retrospective analysis of adult patients with discrete lesions of primary CNS melanoma, Puyana et al. [2] found that patients treated with gross total resection (GTR), GTR paired with radiotherapy (RT), or GTR patient with RT and chemotherapy (ct) had better prognoses than those that had biopsy (Bx) or subtotal resection only (GTR/GTR + ct/GTR + RT > Bx/STR). In these cases, patients with GTR have significantly improved survival than those treated with subtotal resection (STR) (46.3 ± 92.5 months vs. 6 months) or biopsy (1.5 ± 3 months). Balakrishnan et al. [5] reviewed the adult literature and suggested addition of intrathecal methotrexate with or without dexamethasone to craniospinal radiation may be of benefit. Baumgartner et al. [6] reported 26 cases of primary diffuse leptomeningeal melanomatosis in the literature, including adult and pediatric cases. Most patients were treated with chemotherapy (*n* = 10), followed by immunotherapy (*n* = 5) and radiotherapy (*n* = 5). None of the patients survived beyond 12 months, nor was there any clear benefit for a particular treatment approach. Given the limitation in the number of cases, there is no universal consensus on the recommended therapy for these patients. 

Ventriculoperitoneal shunts are utilized in some cases to alleviate intracranial pressure in primary CNS tumors; however, they can lead to the development of peritoneal metastases. In pediatrics, many of these cases are medulloblastoma, high-grade embryonal or glial tumors [18]. In the case of meningeal melanocytic neoplasms, Hironaka et al. [19] reported that all prior cases (*n* = 6) of CNS melanoma with VP shunts resulted in peritoneal metastases. Our patient had a VP shunt due to a history of post-hemorrhagic hydrocephalus related to his prematurity, but, ultimately, it led to tumor dissemination. To our knowledge, this is only the fourth case reported of pediatric meningeal melanomatosis that metastasized to the peritoneal cavity through the peritoneal shunt, but it is the first case with a documented response of the metastatic sites to a combination of nivolumab and binimetinib. 

Molecular profiling of the primary tumor revealed an NRAS Q61R mutation, which is present in approximately 90% of NRAS-mutant cases. Approximately 25% of melanomas are NRAS driven, making this the second most common genetic alteration after BRAF [20]. Treatment lags behind that of BRAF mutant patients, and, thus, NRAS mutant melanoma patients typically also have a worse prognosis. Johnson et al. evaluated NRAS-mutant melanoma patients and determined they have elevated expression of PD-L1 when compared to BRAF mutated or wild-type melanoma. This finding has important implications for therapy. Melanoma patients with NRAS mutations had a trend towards improved outcomes to immunotherapy, either at the first line or subsequent lines of therapy, as well as improved progression-free survival and clinical benefit (response plus stable disease lasting ≥ 24 weeks) [21]. We noted that the PD-L1 stain was negative in our patient. Multiple studies have failed to identify a direct correlation between PD-L1 expression level and response to immunotherapy [22,23,24,25]. Several recent studies have also supported alternate assays to predict response [26,27]. In fact, PD-L1 is not recommended to be used as a biomarker to select patients for receiving anti-PD1 therapy in melanoma [28].

Clinical trials evaluating novel MEK inhibitors as well as combination of these drugs with cyclin-dependent kinase 4/6 (CDK4/6) inhibitors, autophagy inhibitors, pan-RAF inhibitors, and others are underway [20] in localized/metastatic NRAS mutant melanoma. In our patient, the primary tumor revealed an NRAS Q61R mutation while the adult primary CNS melanomas often carry mutations in GNAQ and GNA11. Pedersen et al. [29] argue that NRAS mutations may be the primary drivers in pediatric CNS cases, as a majority of these are associated with giant cutaneous congenital nevi which also tend to carry mutations in NRAS. In a mouse model, they determined that when NRAS G12D mutation was present in congenital nevi, it could induce CNS melanoma, suggesting that this acquired somatic mutation is a potential risk factor for CNS melanoma in children.

Immunotherapy has been an effective strategy for extracranial melanoma [30] as well as for patients with intracranial metastases [25,31]. In the case of primary CNS melanoma, the use of immunotherapy has been limited to a few case reports, and responses have thus far been mixed. El Habnouni et al. [32] treated a 69-year-old woman for 16 weeks with pembrolizumab, but the patient had continued tumor progression. In contrast, Krpan et al. [33] report the case of a 72-year-old patient who experienced 2 years of stable disease following treatment with pembrolizumab. Baumgartner et al. [6] treated a 14-year-old male with nivolumab and ipilimumab. The patient had a complete response for a brief period of time, but then he had rapid progression and died within a few months. 

In contrast to Baumgartner and colleagues, our patient maintained response in brain and spine from radiation and immunotherapy, but he did not experience a response in the abdomen until binimetinib was added to more frequent and higher doses of nivolumab. While Baumgartner et al. used trametinib, we chose to use binimetinib based on available data from adult melanoma trials [34] and the National Comprehensive Cancer Network (NCCN) guidelines. 

Our patient experienced minimal toxicity from his treatment. His only reported toxicity from radiation was mild dermatitis (faint erythema and mild desquamation). Toxicities were grade 2 or less according to Common Terminology Criteria for Adverse Events (CTCAE) 5.0 from the immunotherapy or binimetinib treatments. In the landmark Checkmate-067 trial, 59% of adult melanoma patients treated with a combination of nivolumab and ipilimumab experienced grade 3 or 4 treatment-related adverse events (AEs). The most common AEs were elevated lipase, diarrhea, liver transaminitis, and colitis [35]. In our patient, the infectious complications that led to his death did not appear related to the immunotherapy or MEK inhibitor. 

## 4. Conclusions

This case illustrates the challenges of treating a child with a rare and aggressive CNS tumor with underlying significant medical complications along with the presence of VP shunt that seemingly led to early disease dissemination to the abdomen. Treatment options for children with diffuse leptomeningeal CNS melanomatosis are limited due to the rarity of these cases and lack of clinical trials in pediatric patients. Although our patient ultimately died from an infectious complication and subdural hemorrhage, he had improvement of the CNS disease burden after radiation therapy and demonstrated partial response at the metastatic site following combination treatment with nivolumab and binimetinib. It is also encouraging that despite its rarity, researchers have been able to establish an animal model of this disease [29], which potentially will lead to improved understanding of the pathobiology of this disease, alternative treatment options, and improved outcomes for these patients.

## Figures and Tables

**Table 1:** Primary meningeal melanomatosis cases.

Age (in Years)	Sex (M/F)	Surgery	XRT	CTX	IMMN	Best Response	Time to Death following Diagnosis	Reference
2	F	Biopsy and VP shunt	Y	Temozolomide, cisplatin, vindesine	peg-inter-feron α2b	SD	11 months	Angelino et al. [7]
3	M	Biopsy and V-C shunt	N	N	N	PD	Unknown	Flodmark et al. [8]
5	M	Biopsy	N	vincristine, carboplatin, etoposide	N	PD	6 months	Makin et al. [9]
5	F	Biopsy, multiple cyst decompressions and shunts	N	Cisplatin, etoposide, and temozolomide	N	PD	10 months	Szathmari et al. [10]
5	M	Biopsy and VP shunt	N	Following regimen for PNET at that time in the UK	N	?	Unknown (at least 3 months)	Nicolaides et al. [11]
11	F	Subtotal resection	N/A	N/A	N/A	N/A	2 months	Tanaka et al. [12] *
13	M	Resection of temporal and parietal lesions	N	N	N	PD	5 months	Xu et al. [13]
14	M	Ommaya	Y focal RT (spine)	Trametinib, everolimus (po), etoposide, cytarabine, topotecan (IVT)	Nivolumab, ipilimumab	CR	7 months	Baumgartner et al. [6]
16	F	VP shunt	Y	?	N	?	18 months	Gattuso et al. [14]
17	M	Biopsy	Y Whole brain	Dacarbazine, carmustine, cisplatin, and tamoxifen	N	PR	Unknown (but tolerated 8 cycles)	Lee et al. [15]

M: male; F: female; XRT: radiation; CTX: chemotherapy; IMMN: immunotherapy; IVT: intraventricular; po: oral; V-C: ventriculo-cisternal; PD: progressive disease; SD: stable disease; PR: partial response; CR: complete response; PNET: primitive neuroectodermal tumor; N/A: not applicable; Y: yes; N: no. * In Japanese.

## Data Availability

The data presented in this study are available on request from the corresponding author.

## Supplementary Materials

The following supporting information can be downloaded at: https://www.mdpi.com/article/10.3390/curroncol31010041/s1, Table S1: Case Timeline.
