# Supplementary material for: Primary Diffuse Leptomeningeal Melanomatosis in a Child with Extracranial Metastasis: Case Report
Source: Curr Oncol. 2024 Jan 20;31(1):579–87. doi: 10.3390/curroncol31010041 (PMC10814890; doi:10.3390/curroncol31010041)
Supplement: Supplementary file 1 [file curroncol-31-00041-s001.zip › curroncol-2808564-supplementary.pdf]

Supplemental Table S1. Case Timeline

	Diagnosis	End radiation	3 months	Time of death
Location	Brain and spine<br><br>Peritoneal implants by PET	Brain/spine: Improvement of disease burden; Peritoneal disease + ascites: Progression	Brain/spine: Improvement of disease burden; Peritoneal disease + ascites: Progression by CT and PET	Brain/spine: Improvement of disease burden; Peritoneal disease: improvement; Ascites: improvement by CT
Treatments	Craniospinal radiation	Started nivolumab + ipilimumab	Started higher dose nivolumab+ binimetinib	
